# Positive selection at high temperature reduces gene transcription in the bacteriophage ϕX174

**DOI:** 10.1186/1471-2148-10-378

**Published:** 2010-12-03

**Authors:** Celeste J Brown, Luyi Zhao, Kelsie J Evans, Dilara Ally, Amber D Stancik

**Affiliations:** 1 Department of Biological Sciences, PO Box 443051, University of Idaho, Moscow, ID 83843-3051 USA; 2 Department of Biology, San Diego State University, MC4614, 5500 Campanile Drive, San Diego, CA 92182 USA

## Abstract

**Background:**

Gene regulation plays a central role in the adaptation of organisms to their environments. There are many molecular components to gene regulation, and it is often difficult to determine both the genetic basis of adaptation and the evolutionary forces that influence regulation. In multiple evolution experiments with the bacteriophage ϕX174, adaptive substitutions in *cis*-acting regulatory sequences sweep through the phage population as the result of strong positive selection at high temperatures that are non-permissive for laboratory-adapted phage. For one *cis*-regulatory region, we investigate the individual effects of four adaptive substitutions on transcript levels and fitness for phage growing on three hosts at two temperatures.

**Results:**

The effect of the four individual substitutions on transcript levels is to down-regulate gene expression, regardless of temperature or host. To ascertain the conditions under which these substitutions are adaptive, fitness was measured by a variety of methods for several bacterial hosts growing at two temperatures, the control temperature of 37°C and the selective temperature of 42°C. Time to lysis and doublings per hour indicate that the four substitutions individually improve fitness over the ancestral strain at high temperature independent of the bacterial host in which the fitness was measured. Competition assays between the ancestral strain and either of two mutant strains indicate that both mutants out-compete the ancestor at high temperature, but the relative frequencies of each phage remain the same at the control temperature.

**Conclusions:**

Our results strongly suggest that gene transcription plays an important role in influencing fitness in the bacteriophage ϕX174, and different point mutations in a single *cis*-regulatory region provided the genetic basis for this role in adaptation to high temperature. We speculate that the adaptive nature of these substitutions is due to the physiology of the host at high temperature or the need to maintain particular ratios of phage proteins during capsid assembly. Our investigation of regulatory evolution contributes to interpreting genome-level assessments of regulatory variation, as well as to understanding the molecular basis of adaptation.

## Background

Surveys of variation in gene regulation among different species, populations and individuals indicate that such variation may be strongly influenced by genetic drift and purifying selection [1-4]. This evolutionary pattern is reminiscent of the generally neutral evolution of synonymous sites and of purifying selection on non-synonymous sites in protein-coding genes [5]. Similar to positive selection observed at non-synonymous sites in protein-coding genes, changes in gene expression also have been shown to be subject to positive selection [6-8]. Investigating genes whose expression is affected by positive selection will allow us to understand the role of gene regulation in evolution [9-12].

The distinction between the molecular components that influence gene regulation that might evolve in a neutral fashion and those that may respond to positive or purifying selection is not clear [13-15]. Indeed, only a few studies have shown the adaptive value of point mutations in c*is*-acting regulatory sequences or *trans*-acting regulatory proteins let alone the effect of such adaptive substitutions upon gene regulation [16-19]. Additionally, genetic variation within promoter regions may affect chromatin structure leading to differences in gene expression [18]. Furthermore, c*is*-regulatory sites and *trans*-regulatory proteins may occur in a variety of combinations with the same effect on gene expression [20]. This complexity makes it difficult to determine the genetic basis of regulatory variation or the forces that influence their evolution.

A reductionist approach in which adaptive substitutions in regulatory regions are tested for their influence on gene regulation and fitness provides another pathway for evaluating the role of gene regulation in evolution [4]. First, the adaptive substitution and its role in gene regulation as either a *cis*- or a *trans*-regulatory mutation are known. Second, the evolutionary forces that led to the substitution's detection are clearly defined by the experimental conditions under which it was discovered. Third, the effects of the substitution on gene regulation and on fitness are easily determined. In this approach, simple hypotheses can be easily defined and tested. For example, mutations that alter transcription factor binding sites such that binding efficiency is reduced are hypothesized to lead to the down regulation of gene transcription, but the environments in which such down regulation will improve fitness are unknown. These points can be addressed directly by a reductionist approach, providing a bottom up view of the evolutionary forces and genetic basis of regulatory variation.

Such an approach requires a model organism whose biology and evolution are well studied. For over 50 years, the bacteriophage ϕX174 has been a model organism in the study of DNA replication, gene regulation and virion structure, so that the molecular biology and physiology of this positive-strand, ssDNA phage is well understood [21,22]. ϕX174 utilizes its host's replication, transcription and translation machinery in order to reproduce making it an amenable organism for studies of regulatory evolution. In particular, the only components of the gene expression machinery that are encoded by the phage genome are the *cis*-acting regulatory sequences, although a possible role for the phage protein H was recently proposed [23]. Hence, studies of regulatory evolution using ϕX174 can focus on the importance of *cis*-acting sequences.

**Figure 1:**
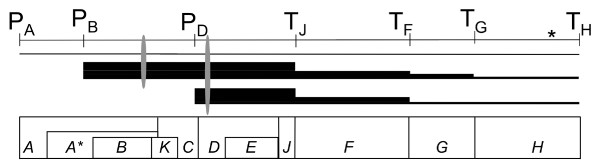
**Regulation of ϕX174 gene transcription**. Gene transcription in ϕX174 is regulated by four promoters (P_A_, P_B_, P_D_, *) and four transcription terminators (T_J_, T_F_, T_G_, T_H_). Bars indicate transcript start and stop and thickness of bars indicates relative amount of transcript. Ovals show approximate positions of sequences detected in qPCR experiments. Gene locations are shown below, and genes *B, K *and *E *are in different reading frames from the genes with which they overlap. The ϕX174 genome is a circular molecule; the figure was linearized for clarity. From [21,22].

The simplicity of ϕX174 gene regulation also makes it a useful model for evolutionary studies of gene transcription. Over the approximately 12 minute life cycle of the wild type phage, 11 genes are expressed (Figure 1, bottom). These genes encode six procapsid proteins (B, D, F, G, H, J), two proteins that are involved in viral genome replication (A, C), two that interact with other phage or the host (A*, E), and one whose function is unknown (K) [22,24]. Gene transcription is controlled by promoters that appear to consist solely of sigma factor binding sites upstream of genes *A, B *and *D *(Figure 1, top; P_A_, P_B_, P_D_) [25-27]. The transcript that starts from P_A _encodes the A and A* proteins and is very unstable. The length of transcripts starting from P_B _and P_D _varies based upon which of the rho-independent termination signals after genes *J, F, G *and *H *are used (Figure 1, top; T_J_, T_F_, T_G_, T_H_) [28,29]. Thus the transcripts that begin from P_B _and P_D _overlap in encoding six of nine genes (Figure 1, middle). The percentage of transcripts that continue through a termination signal to the following gene differs for each terminator (Figure 1, middle), and there is considerable variation in the half-life of each transcript [28,30]. A ribosome binding site immediately upstream of the start codon has been identified experimentally for genes *A*, *D, E, F, G *and *H*, and there are consensus Shine-Dalgarno sequences appropriately spaced upstream of *B *and *J*, but not before C [27,30,31]. ϕX174 is an excellent model organism given this detail of understanding of its life cycle and the relative simplicity of its regulatory machinery.

For over 15 years, the bacteriophage ϕX174 has been a model organism for the study of the genetic basis of adaptation as well [32]. Many of these studies have grown phage in chemostats under various environmental conditions to observe evolution under strong positive selection with negligible influence of genetic drift [33,34]. Selection pressures in the chemostats include competition between phage and adaptation to novel temperatures and/or hosts, which are not allowed to evolve during these experiments. Multiple parallel substitutions arise under similar environmental conditions, especially in the major capsid protein F, the replication initiation protein A and the pilot protein H [32]. These chemostat experiments also have resulted in adaptive substitutions in the promoters before genes *A, B *and *D*, the ribosome binding sites of *A, E *and *G*, and the transcript termination loops after genes *J *and *H *[HA Wichman, personal communication; [35-38]]. Indeed, the fraction of sites undergoing adaptive substitutions in both regulatory sequences and protein sequences is the same, 11-12%, even though the target size for regulatory substitutions is an order of magnitude smaller than the total protein sequence of this phage [32]. Thus the adaptive substitutions identified in these experiments provide multiple opportunities for understanding the molecular and phenotypic consequences of regulatory evolution.

Our goal is to understand the processes involved in regulatory evolution by identifying adaptive substitutions in regulatory sequences, observing the influence these substitutions have upon gene regulation and correlating this regulatory variation to fitness. Our choice of regulatory substitutions is guided by their spread under experimental conditions that elicit strong positive selection. Therefore, we are focusing on adaptive substitutions that have accumulated in *cis*-regulatory sequences of the bacteriophage ϕX174 in response to selection at high temperatures in previous evolution experiments [32]. In the present study, we find that these adaptive substitutions down-regulate gene expression at both temperatures and that they consistently increase fitness at high temperature, regardless of bacterial host.

## Results

The goal of this study was to determine whether strong positive selection influences gene transcription. In multiple chemostat evolution experiments, adaptive substitutions arose in at least one of the three ϕX174 promoters. We chose to study the -35 region of the sigma factor binding site upstream of gene *D *(Figure 1; P_D_), because substitutions arose in this region in response to selection at high temperature in three hosts, *Escherichia coli, Shigella sonnei *and *Salmonella typhimurium *(Table 1) [HA Wichman, personal communication; [34-38]]. These substitutions arose within 10 days and as early as five days when grown on *S. typhimurium *at high temperature and within 20 days when grown on *E. coli *or *S. sonnei*. In only two experiments were more than one of these four substitutions detected in the population at the same time. Interestingly, in both cases ^*G*^*319*^*T *^was fixed in the population and ^*A*^*323*^*G *^eventually was lost [34,37].

**Table 1:** Characteristics of adaptive substitutions in the D promoter of ϕX174 that arose in previous evolution experiments.

Strain	Mutation	Change in Protein C	# of Exps	Host*	Temperature, °C*
mut319	^*G*^*319*^*T*^	^V^63^F^	4**	*Salmonella typhimurium*	43.5
			2, 1	*Escherichia coli*	37, 43.5
mut321	^*T*^*321*^*C*^	none	1	*Salmonella typhimurium*	43.5
mut323	^*A*^*323*^*G*^	^N^64^G^	3	*Salmonella typhimurium*	43.5
			1	*Escherichia coli*	43.5
mut324	^*C*^*324*^*T*^	none	1	*Shigella sonnei*	42
			1	*Escherichia coli*	43.5

*Conditions under which substitutions arose [HA Wichman, pers. comm.; [35-38]]

** Also, one reversion after switch to *E. coli *host at 43.5°C.

The gene for the DNA packaging protein C is immediately upstream of gene *D *and contains the entire *D *promoter sequence. Two of the substitutions, ^*G*^*319*^*T *^and ^*A*^*323*^*G*^, affect the amino acid sequence of protein C (^V^63^F^, ^N^64^G^, respectively). Two of the substitutions, ^*T*^*321*^*C *^and ^*C*^*324*^*T*^, are silent substitutions. Additionally, ^*G*^*319*^*T *^is immediately adjacent to the canonical -35 sigma factor binding site, rather than within the site, so the effect of nearby substitutions can be observed. Using site-directed mutagenesis of the ancestral ϕX174 sequence, four strains were constructed that had a single substitution at one of these four sites [35]. Quantitative PCR (qPCR) was used to measure the transcript levels from the *D *promoter for the ancestral and mutant strains under each environmental condition. Two fitness assays, time to lysis and doublings per hour, were conducted for each of the four mutant strains and for the ancestral strain at 37°C and 42°C in each of the three bacterial hosts. Chemostats were used to directly compete the ancestor with either of two mutant strains at 37°C or 42°C using *E. coli *C as the host.

### Transcription is strongly down-regulated for all four mutants regardless of temperature or host

The promoter upstream of *B *controls the start of transcription for up to nine of eleven ϕX174 genes, including *C*, and these transcripts overlap with the transcripts for six genes that start at the promoter upstream of gene *D *(Figure 1). Amplification of cDNA for gene *D*, therefore, includes transcripts from both the *B *promoter and the *D *promoter. Thus, the transcript level of the *B *gene can serve as an internal control for the efficiency of infection, for contaminating phage DNA and for the effect of temperature or host on phage transcription in general. Additionally, the transcript levels for gene *B *are a proxy for the transcript levels for gene *C*, because transcripts other than those shown in Figure 1 are rapidly degraded and do not contribute to phage mRNA levels [28].

**Figure 2:**
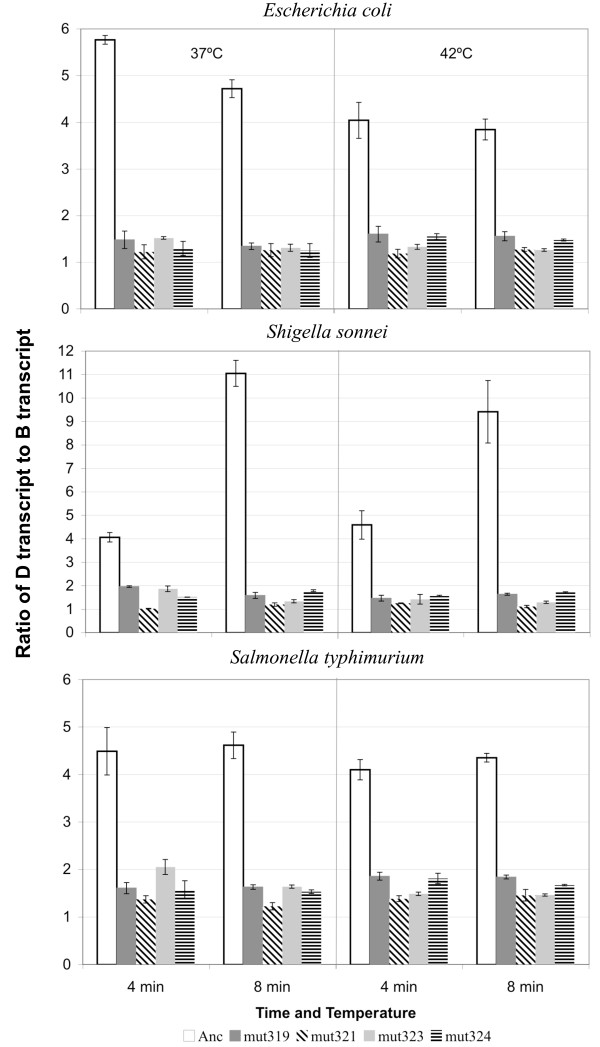
**Transcript levels differ between ancestor and promoter mutant strains at two temperatures in three bacterial hosts**. The amount of transcript starting from the D promoter was measured relative to the amount of transcript starting from the B promoter for each phage strain using quantitative PCR. Injection of DNA into host was synchronized by attachment at 15°C and then addition to LB at 37°C. Samples were taken at 4 and 8 min after injection, which is prior to the start of cell lysis. The y-axis indicates the ratio of the D transcript to the B transcript. Bars are the mean and error bars the standard error of two (*S. sonnei*) or three replicate assays. Solid bars are mutant strains with non-synonymous substitutions in *C*, hatched bars are synonymous in *C*. Relative transcript level of the ancestor is significantly greater than the four mutants for all host/temperature/time combinations (Table 2). Note the difference in scale for the *S. sonnei *graph.

The effect of mutations in the promoter upstream of gene *D *was determined at two time points after the synchronized injection of phage DNA into host cells. After RNA extraction and conversion to cDNA, regions of the *B *and *D *cDNAs (Figure 1, grey ovals) were amplified by qPCR, and the ratio of the *D *transcript relative to the *B *transcript was measured (Figure 2). In each host at both temperatures and at both time points, the consequences of the mutations in the promoter were the same. All of the mutant strains were significantly down-regulated compared with the ancestor (Table 2). The decrease in the amount of transcript for the *D *gene ranged from no transcript to almost the same amount of transcript as for the *B *gene.

**Table 2:** Transcript level from the D promoter is significantly reduced in promoter mutant strains at any temperature, host or time point*.

Temperature	Host	Time	F-test	Pr > F
37°	*Escherichia coli*	4 min	395	<0.0001
37°	*Escherichia coli*	8 min	397	<0.0001
37°	*Salmonella typhimurium*	4 min	53	<0.0001
37°	*Salmonella typhimurium*	8 min	209	<0.0001
37°	*Shigella sonnei*	4 min	450	<0.0001
37°	*Shigella sonnei*	8 min	1112	<0.0001
42°	*Escherichia coli*	4 min	143	<0.0001
42°	*Escherichia coli*	8 min	390	<0.0001
42°	*Salmonella typhimurium*	4 min	381	<0.0001
42°	*Salmonella typhimurium*	8 min	473	<0.0001
42°	*Shigella sonnei*	4 min	94	0.0002
42°	*Shigella sonnei*	8 min	143	<0.0001

*ANOVA on relative transcript levels for each environmental condition and time point with 1 degree of freedom for each test.

### Fitness of all mutant strains was higher than ancestor at high temperature

**Figure 3:**
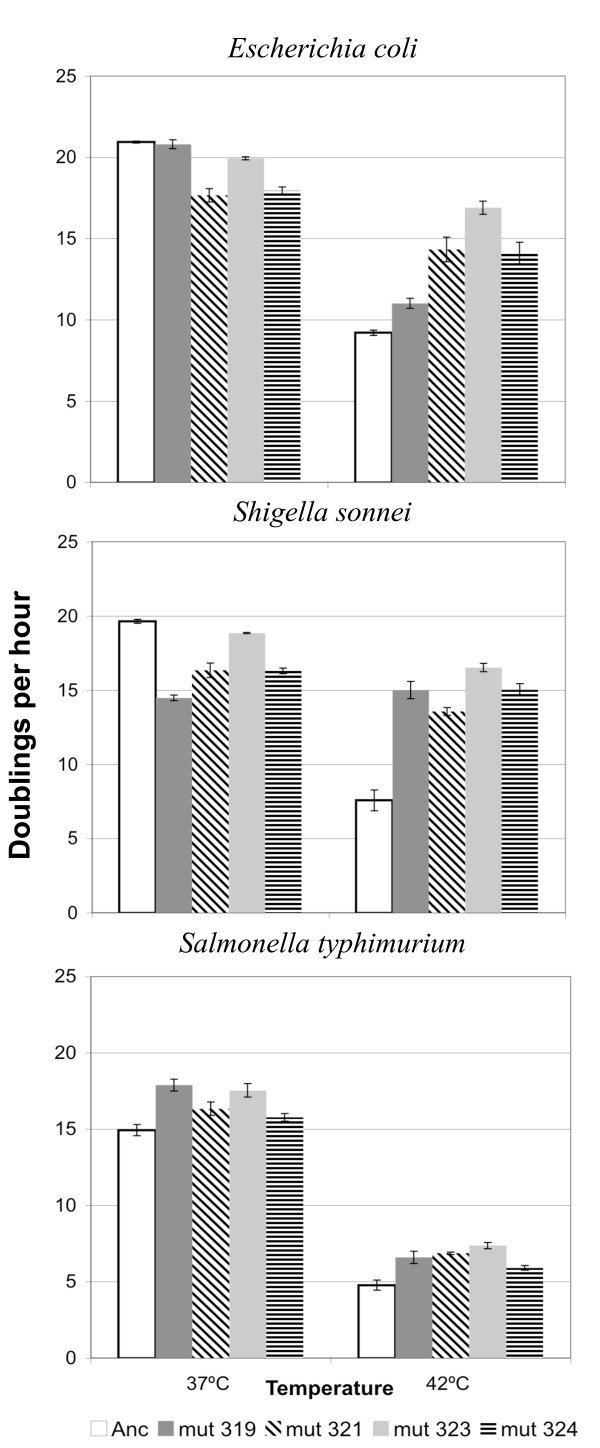
**Fitness differences between ancestor and promoter mutant strains at two temperatures for three bacterial hosts**. Doublings per hour were measured for each phage strain in three bacterial hosts and at two temperatures. Bars are the mean and error bars the standard error of three replicate assays. Solid bars are mutant strains with non-synonymous substitutions in *C*, hatched bars are synonymous in *C*. Doublings per hour of the ancestor is significantly lower than at least one of the four mutant strains for all host/temperature combinations except *E. coli *and *S. sonnei *at 37°C, where the ancestor's fitness is significantly greater than at least one of the four mutant strains (Table 3).

Fitness was measured as the number of times the phage population size doubles in an hour when grown in batch culture. Fitness was measured for each strain with each host at each temperature, and the fitnesses of the mutant strains were compared to that of the ancestor. In all three hosts, the mutants had more doublings per hour than the ancestor when grown at 42°C regardless of the host, but the amount of improvement of the mutant over the ancestor at 42°C depended upon the host (Figure 3, Table 3). At 37°C, relative fitnesses of mutants to ancestor depended upon the host. In *S. typhimurium*, all of the mutants had higher fitness than the ancestor (p = 0.001). In *S. sonnei*, all of the mutants had lower fitness (p < 0.0001); in *E. coli*, the mutants had the same or lower fitness than the ancestor at 37°C (Table 3).

**Table 3:** Fitness of promoter mutants is greater than ancestor at 42°C*.

Temperature	Host	F-test	Pr > F	t-value	Pr > |t|
37°	*Escherichia coli*	41.4	<0.0001	6.7	<0.0001
37°	*Salmonella typhimurium*	10.6	0.0013	-4.6	0.0010
37°	*Shigella sonnei*	70.6	<0.0001	11.3	<0.0001
42°	*Escherichia coli*	35.4	<0.0001	-8.6	<0.0001
42°	*Salmonella typhimurium*	14.8	0.0003	-6.6	<0.0001
42°	*Shigella sonnei*	53.2	<0.0001	-13.9	<0.0001

*ANOVA on doublings per hour for each environmental condition with 4 degrees of freedom for each test, and t-test comparing ancestor to all mutants.

### Mutants out-compete the ancestor in a chemostat at high temperature

**Figure 4:**
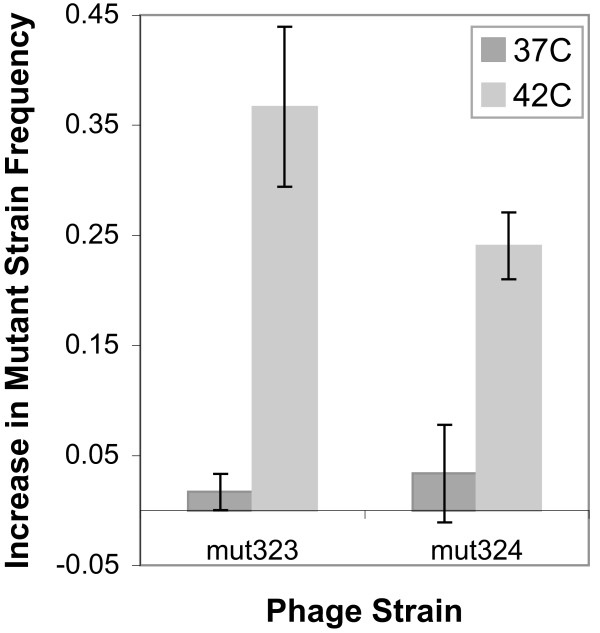
**Competition assays confirm the greater fitness of mutant strains at 42°C in *E. coli***. The ancestor and one mutant strain were grown together in a two-stage chemostat on *E. coli *C at 37°C or 42°C. Concentration of each strain was determined by allele specific PCR at the beginning and end of each chemostat. The y-axis shows the average increase in frequency of the mutant strain after two hours. Three replicate chemostats were run for each mutant at each temperature. Mut323 is a non-synonymous substitution in *C*, and mut 324 is synonymous. The increase in mutant strain frequency was significantly different between the two temperatures for both mutants (Wilcoxon rank sum test, p < 0.005).

The substitutions in the *D *promoter evolved in the high multiplicity of infection (MOI) environment of a chemostat, but the fitness assays were conducted in low MOI batch cultures. Competition assays between the ancestor and either of two mutant strains were conducted in chemostats to confirm that the differences in fitness shown in Figure 3 reflect a competitive advantage within a chemostat. Figure 4 shows the average change in frequency of the mutant for replicate competition experiments in *E. coli *at two temperatures. At 37°C, the frequency of the mutant barely changes, even when the chemostat is started at different ratios of mutant to ancestor. At 42°C, however, the mutant frequency increases to around 80% over a two-hour experiment (Figure 4). In contrast with the low MOI fitness measurements, which show that doublings per hour of the mutant is less than the ancestor, the competition assays at 37°C indicate that the ancestor does not out-compete the mutants. The results of the competitions at 42°C, however, coincide with the low MOI fitness measurements. The change in frequency of the mutant strains in the 42°C chemostats is significantly greater than in the 37°C chemostats (Wilcoxon rank sum test, p < 0.005).

### Time to lysis for all mutant strains is different from ancestor, except in *S. typhimurium*

One component of overall fitness is the time it takes from injection of the phage DNA into the cell until the cell is lysed, releasing the progeny phage. Phage that lyse earlier can infect new hosts earlier, but late-lysing phage may have more progeny. Time to lysis was measured for each of the five phage strains under six conditions: three hosts at two temperatures. The time point at which the number of phage in the culture was greater than or equal to twice the number at the zero time point determined time to lysis. This is a coarse-scale assessment because time points were taken every two minutes starting at ten minutes after eclipse of the phage into the host cell. This coarse scale was more than sufficient for seeing differences between phage in *E. coli *and *S. sonnei*. In both hosts, the ancestor lysed by 12 minutes at 37°C and by 14 minutes at 42°C. Each of the mutants, on the other hand, lysed by 14 minutes at 37°C and by 12 minutes at 42°C, reversing the pattern of the ancestor. This pattern was not seen in *S. typhimurium*, where only mut323 at 42°C showed an earlier lysis time of 14 minutes. All of the other phage lysed *S. typhimurium *by 16 minutes at both temperatures.

## Discussion

We have shown that substitutions in regulatory sequences contribute to adaptation under strong selective conditions. These adaptive substitutions down-regulate the expression of transcripts from the *D *promoter. Compared to the ancestor, the mutants had significantly lower transcript levels regardless of the host or temperature at which the phage were propagated. This change in expression, however, was only consistently correlated with adaptation in the high temperature conditions under which these substitutions were first identified.

One reason for choosing the -35 sequence of the *D *promoter was because of the number of sites at which adaptive substitutions arose in this region and because two of the four substitutions were synonymous, ^*T*^*321*^*C *^and ^*C*^*324*^*T*^, and two non-synonymous, ^*G*^*319*^*T *^and ^*A*^*323*^*G*^, in protein C (Table 1). Thus, we are able to observe the fitness effect of the changes in transcription alone and the changes in transcription and in the amino acid sequence of C. The two synonymous substitutions appear to have almost identical doublings per hour for each experimental condition (Figure 3). The two non-synonymous substitutions, on the other hand, do not appear to be similar to each other or to the synonymous substitutions. There appear to be pleiotropic effects on fitness due to the amino acid substitutions in protein C for the two non-synonymous substitutions, as we expected.

Although synonymous substitutions are known to be adaptive under certain circumstances, these circumstances are not applicable here. The synonymous substitutions, ^*T*^*321*^*C *^and ^*C*^*324*^*T*^, are for common codons in the *E. coli *C genome. Moreover, the correlation between codon bias and selective advantage is generally correlated with highly-expressed genes, and protein C has a very low level of expression [39]. Indeed, there is no canonical Shine-Dalgarno sequence before C's translation start codon. It is possible that all four substitutions affect some other aspect of phage biology, such as genome packaging or DNA secondary structure, however, the inconsistency in the effect on GC-content suggests that these are also unlikely factors. The strict decrease in transcript levels for all conditions and the significant increase in fitness at high temperature for all hosts suggests to us that transcription provides the underlying basis for the adaptive advantage of these synonymous substitutions.

It was somewhat surprising to us that each of the four substitutions would have such a strong effect on fitness on their own because we expected epistatic effects to be important. ϕX174 was lab-adapted to *E. coli *starting in the 1950's, and its fitness on *S. typhimurium *is quite low (Figure 3). Multiple experiments have shown that the first adaptive substitutions to sweep through a chemostat in which ϕX174 is grown on *S. typhimurium *at 43.5°C generally are in the capsid protein F, and these substitutions seem to affect capsid stability and/or host attachment [38,40]. These selective sweeps are accompanied by a large increase in the fitness of the evolved phage. Either the ^*G*^*319*^*T *^or the ^*A*^*323*^*G *^substitutions were subsequently seen in these same populations, and ^*G*^*319*^*T *^and several of the adaptive substitutions in the capsid protein reverted to the ancestral state when the strain carrying them was grown on *E. coli *[36,37]. These results suggest that the amino acid substitutions in protein C (^V^63^F^, ^N^64^G^) are interacting either with the host or with the capsid protein. The small increase in fitness for our single mutants when *S. typhimurium *was the host (Figure 3) further suggests that the interaction is with the *Salmonella*-adapted capsid protein. We hypothesize that had adaptive substitutions been present in the capsid proteins of the mutant strains, the increase in fitness due to the regulatory substitutions would have been more substantial. In particular, because ^*G*^*319*^*T *^has occurred in the most high temperature, *Salmonella *experiments, we expect that it has the greatest fitness effect in a *Salmonella*-adapted background contrary to its small fitness advantage relative to the other mutants. Tests for such epistatic and pleiotropic interactions await future experiments.

We can think of several non-exclusive hypotheses to test our conclusion that down regulating gene expression is adaptive at high temperature. First, the higher temperature is deleterious for the host as well as the phage. The main costs of constitutive expression of non-essential genes in *E. coli *are the acts of transcription and/or translation, and over expression of genes is also costly in yeast [41,42]. Since the phage use the transcription and translation machinery of the host, down regulating gene expression may reduce the stress on the host's regulatory machinery, thus maximizing the number of phage progeny produced.

Secondly, it seems reasonable to predict that the ratio of the different transcripts (Figure 1) reflects the levels needed to produce the optimal ratio of proteins for each capsid. The ϕX174 procapsid is composed of 60 B (internal scaffold), 60 F (major capsid), 60 G (major spike), 12 H (pilot) and 240 D (external scaffold) proteins. In the provirion, the B proteins are replaced by 60 J (DNA binding) proteins [21]. The reduction in transcript levels due to the three transcription terminators prior to gene *H*, which has the lowest number of subunits per procapsid, and the strong promoter before gene *D*, which has the highest number of subunits per procapsid, lend support for this prediction (Figure 1). Environmental changes that disrupt the ratio of phage proteins, such as increased temperature, may be a selective force leading to changes in gene regulation.

Our second hypothesis, therefore, is that the relative concentrations of proteins at high temperature are suboptimal, possibly because some of the proteins are unstable or because the efficiency of the transcription terminators changes at the higher temperature. Protein B is required for the assembly of pentamers of protein F, for the assembly of pentamers of protein G and pentamers of protein F into an intermediary complex, and B facilitates the incorporation of protein H into this complex [43,44]. Protein B appears to have a chaperone-like quality in that it keeps protein F as well as protein H from aggregating [23,45]. If the amount of protein B that is produced at 42°C is insufficient for performing these functions, then decreasing the amount of proteins F and H by down regulating transcription from the *D *promoter may optimize the ratio of phage proteins at this temperature. Our future work will test whether maintaining the proper ratio of phage proteins is the molecular basis of these adaptive regulatory substitutions.

These two hypotheses do not shed light on the puzzling inconsistency between the time to lysis studies and the lower transcription rates of the mutants. The lysis protein E is a transmembrane protein that affects the host so that at cell division, the cell membrane fails and lysis occurs [46]. It seems consistent that at 37°C, the ancestor would lyse earlier than the mutants, which are producing less transcript from the *D *promoter, and hence, fewer transcripts for protein E (Figure 1). It is not consistent that at 42°C when the transcription level of the mutants is again low relative to the ancestor, that the time to lysis should decrease for the mutants. Our first hypothesis seems to be the most consistent with the results at 42°C. If the host's transcription/translation machinery are overtaxed at high temperature, the length of the cell cycle may be increased. Decreasing the pressure on this machinery by down regulating gene expression may lead to earlier cell division, and thus earlier lysis.

## Conclusions

One consequence of strong positive selection on the bacteriophage ϕX174 was to down regulate gene transcription from one of two promoters whose transcripts overlap in encoding six of nine genes. The fitness advantage that correlated with this change in transcript levels was consistent only under high temperature conditions, suggesting that understanding the evolution of gene regulation will rely upon evaluating regulatory variants under the appropriate environmental conditions. The physiological mechanism by which decreased transcript levels may lead to increased fitness is still to be ascertained. Continued in-depth studies on the molecular basis of adaptation by changes in gene regulation will provide essential background for following up on whole-genome expression studies.

## Methods

### Phage and Hosts

A wild-type isolate of ϕX174 was used as the ancestor for these studies (GenBank Accession AF176034). The hosts used for phage propagation were *Escherichia coli *C, *Salmonella typhimurium *LT2 strain IJ750 [*xyl-404 metA22 metE551 galE719 trpD2 ilv-452 hsdLT6 hsdSA29 hsdSB1 fla-66 rpsL120 H1-b H2-c nix*], and *Shigella sonnei *strain NCDC 1120-66 [CIP 104223]; (ATCC #25931).

### Site-directed Mutagenesis

Four mutant strains were constructed in the ancestral background using site-directed mutagenesis. Each half of the phage was amplified using Platinum Pfx DNA Polymerase from Invitrogen (Cat. No. 11708) following the manufacturer's directions. The forward mutant primers were paired with 2953R and the reverse mutant primers were paired with 2605F in separate reactions (Table 4). The two products for each mutant were combined in another PCR to join the two halves, and this reaction was electroporated into competent *E. coli *C cells. The electroporated cells were plated and plaques were purified by a second round of plating. The entire genomes of each purified plaque were sequenced to confirm the presence of the desired mutation and no others. Each mutant strain had a single mutation in the *D *promoter: mut319 (^*G*^*319*^*T*^), mut321 (^*T*^*321*^*C*^), mut323 (^*A*^*323*^*G*^), or mut324 (^*C*^*324*^*T*^).

**Table 4:** Oligonucleotides used for site-directed mutagenesis, allele-specific PCR and quantitative PCR

Primer Name	Location*	Sequence (5' to 3')	Purpose
319F	314-338	CTCTTTTTGACATTTTAAAAGAGCG**	Mutagenesis
319R	338-314	CGCTCTTTTAAAATGTCAAAAAGAG	Mutagenesis
321F	314-338	CTCTTGTCGACATTTTAAAAGAGCG	Mutagenesis
321R	338-314	CGCTCTTTTAAAATGTCGACAAGAG	Mutagenesis
323F	314-338	CTCTTGTTGGCATTTTAAAAGAGCG	Mutagenesis
323R	338-314	CGCTCTTTTAAAATGCCAACAAGAG	Mutagenesis
324F	314-338	CTCTTGTTGATATTTTAAAAGAGCG	Mutagenesis
324R	338-314	CGCTCTTTTAAAATATCAACAAGAG	Mutagenesis
2605F	2605-2626	CAGGTTGTTTCTGTTGGTGCTG	Amplification
2953R	2937-2953	CCGCCAGCAATAGCACC	Amplification
Anc_asF	305-324	GTAGAGATTCTCTTGTTGAC	Allele-specific
mut323_asF	305-323	GTAGAGATTCTCTTGTTGG	Allele-specific
mut324_asF	304-324	GGTAGAGATTCTCTTGTTGAT	Allele-specific
phiXpD_asR	625-603	GCAATAAACTCAACAGGAGCAGG	Amplification
phiXpB_F	5337-5360	CTCAAATTTATGCGCGCTTCGATA	qPCR Primer
phiXpB_M	5377-7	CCAACCTGCAGAGTTT	qPCR Probe
phiXpB_R	27-7	TTCTGCGTCATGGAAGCGATA	qPCR Primer
phiXpD_F	400-424	TTACTGAACAATCCGTACGTTTCCA	qPCR Primer
phiXpD_M	427-443	CCGCTTTGGCCTCTATT	qPCR Probe
phiXpD_R	466-457	ACGGCAGAAGCCTGAATGAG	qPCR Primer

* Based upon Sanger, *et al. *sequence [24].

** Mutation underlined

### RNA Sampling and Purification

To synchronize injection of the phage into the host cells, 1.5 × 10^8 ^cells were incubated with 1.5 × 10^7 ^phage in a 1 ml eppendorf tube at 15°C (10°C for *Salmonella*) for one hour. The cells and phage were centrifuged in a benchtop micro-centrifuge at 13000 rpm for 5 minutes at 4°C. The supernatant containing unattached phage was discarded and pellets were re-suspended in 1 ml of ice cold LB + 2 mM CaCl_2_. The resuspended pellet was added to 9 mls of LB + 2 mM CaCl_2 _pre-equilibrated at either 37°C or 42°C; this procedure brings the culture above 22°C, at which point attached phage inject their DNA into the host. After the attached phage and cells were added to the temperature-equilibrated media, four and eight minute samples of 500 μl were added directly to 1 ml of RNAprotect™ Bacteria Reagent (Qiagen Cat. No.76506) and incubated at room temperature for 5 minutes to stabilize the RNA. The samples were centrifuged for 10 minutes in a micro-centrifuge at room temperature, and total RNA was purified using the RNeasy Mini Kit (Qiagen Cat. No.74104). The optional on-column DNase digestion using the RNase-Free, DNase Set (Qiagen Cat. No. 79254) was performed on filter following the guidelines in the Qiagen protocol to remove contaminating DNA.

### qPCR Primers and Probes

Primers and 5' FAM™ dye-labeled Taqman^® ^MGB probes with non-fluorescent quencher dyes were designed and optimized by Applied Biosystems (Custom Taqman^® ^Gene Expression Assays Part No. 4331348) using their Custom Taqman^® ^Assay Design Tool available on their website. The primer and probe sets detected cDNAs for genes *B*, nt 5075-49, or *D*, nt 390-848 (Table 4). They were tested for amplification efficiency using five 10-fold dilutions of the cDNA to produce a PCR standard curve, and the C_T _values were plotted against log input nucleic acid mass. A 100% efficient reaction will yield a 10-fold increase in PCR amplicon every 3.32 cycles during the exponential phase (Applied Biosystems protocol). Two dilutions closest to 100% efficiency, 10^-1 ^and 10^-2^, were used in all subsequent experiments. Since *E. coli *genomic DNA and RNA are present in the samples, we tested the primers and probes designed for genes *B *and *D *of the bacteriophage for amplification of purified *E. coli *DNA. We also tested the cDNA samples purified with only bacteria in the sample for the same reason, and no amplification was found in either case.

### Reverse Transcription and Real-time PCR

Reverse transcription was carried out with High-Capacity cDNA Reverse Transcription Kit (Applied Biosystems #4368813) according to the manufacturer's protocol. The amplification and detection of the cDNA were carried out using Applied Biosystems 7900HT Fast Real-Time PCR Sequence Detection System in a 96-Well Optical Reaction Plate (Applied Biosystems). Thermal cycling conditions started with 95°C hold for 10 minutes followed by 40 cycles of 95°C for 15 seconds then 60°C for 1 minute. For determining the C_T _values, the baseline was set to automatic and the threshold values were all manually set at 0.2. Positive controls used each set of primers against the same preparation of phage DNA. Negative controls included using each set of primers on the RNA to test for contaminating DNA and on a no target reaction mix. The ratio of D transcript relative to the B transcript was determined by raising 2 to the difference between the C_T _values for the B and D transcripts.

### Fitness Assay

Growth rate was tested as an index for fitness for each strain [34]. Host cells were grown to a density of ~2 × 10^8 ^cells/mL by shaking at 37°C in 125 ml flasks with 10 mL LB supplemented with 2 mM CaCl_2_. The cultures were moved to the experimental temperature, and phage were added at an initial concentration of ~10^4^/mL. Samples were titered at two time points, 0 min and 40 min. Growth rate in doublings per hour were calculated as: [log_2_(phage concentration at t = 40) - log_2_(phage concentration at t = 0)](60 min/40 min).

### Chemostat Competition Assay

The ancestral strain was allowed to compete with either of two mutant strains, mut323 or mut324, in a two-stage chemostat. The chemostat consisted of 2 100 × 15 mm glass test tubes. The first tube contained *E. coli *C with a continuous supply of LB broth containing 0.005% antifoam B (Astoria-Pacific Product # 90-0703-01) to maintain cell growth. The mixture in the cell tube was drawn into the second tube that contained the phage. Approximately 2 mls was maintained in each tube with a flow rate of approximately 9 ml/hr. The *E. coli *was grown in LB at 37°C for one hour with shaking prior to addition to the chemostat. Four mls of these cells were added to the cell tube and allowed to flow into the phage tube. The amount was allowed to level out at 2 mls once again. One of the mutants and the ancestor were added to the chemostat at various concentrations (10^8 ^total phage) with a syringe, and the chemostat was run at either 37°C or 42°C for two hours. The chemostat was run for two hours to minimize the possibility of mutations arising and sweeping through the chemostat. Samples were taken every fifteen minutes using a port in the phage tube, and the phage in the chemostat were titered at each of these time points to confirm that no such sweep occurred as indicated by a sudden rapid increase in titer. Allele-specific PCR (asPCR) was used to amplify 20 plaques from the mixture of phage added to the chemostat and 20 plaques from the final chemostat sample to determine the frequency of the ancestor and the mutant strains at the beginning and end of each competition.

### Allele-specific PCR

For each plaque purified from the competition experiments, separate asPCR assays were performed for each phage strain included in the chemostat using primer pairs that specifically amplify only one strain (Table 4). The specificity of each allele-specific primer pair was confirmed for every PCR run by the inclusion of both positive and negative controls. Except for the optimized annealing temperatures, which were 63.1°C for mut323 and mut324 and 63.5°C for Ancestor, PCR was performed using Amplitaq Gold PCR Master Mix (Applied Biosystems) according to the manufacturer's protocol for a three-temperature cycle. PCR products were visualized on agarose gels stained with ethidium bromide and each reaction was scored for presence or absence of amplification.

### Time to Lysis Assay

To find the time to lysis for the ancestor and each of the D promoter mutant strains, the samples were prepared as described for the transcription experiments except that 10^7 ^cells were combined with 10^6 ^phage. After attached phage were brought to the experimental temperature, samples were taken every two minutes for up to 24 minutes, plated on LB agar plates, and the plaques were counted after 3-4 hours of incubation at the experimental temperature. The time point at which the number of phage in the culture was greater than or equal to twice the number at the zero time point determined time to lysis.

### Statistical Analyses

A three-factor ANOVA was conducted on the data for doublings per hour and a four-factor ANOVA was conducted for D transcript level to test whether the mutant strains differed significantly from the ancestral strain. Because the other factors, host, temperature and (time), all showed significant interactions with strain, a one way ANOVA was conducted on each experimental condition separately. The null hypothesis for each combination of host, temperature and (time) was that the four mutant strains did not differ significantly from the ancestral strain.

## Authors' contributions

CJB conceived of the study, participated in its design and coordination and drafted the manuscript. LZ conducted fitness and transcription assays in *Shigella*; KJE conducted fitness assays in *E. coli *and *Salmonella*; DA developed the allele-specific PCR assay; ADS conducted fitness, transcription and competition assays in *Salmonella *and/or *E. coli*. All authors read, provided intellectual input on and approved the final manuscript.
